# A Case Report of an Unusually Large Heterotopic Pancreas

**DOI:** 10.7759/cureus.64228

**Published:** 2024-07-10

**Authors:** Cindy C Iwuagwu, Lillie Jensen, Jignesh Parikh, Vania Zayat

**Affiliations:** 1 Internal Medicine, University of Central Florida College of Medicine, Orlando, USA; 2 College of Medicine, University of Central Florida College of Medicine, Orlando, USA; 3 Pathology, Orlando Veterans Affairs Medical Center, Orlando, USA; 4 Pathology, University of Central Florida College of Medicine, Orlando, USA

**Keywords:** gastrointestinal nodules, ectopic pancreas, pathology, internal medicine, gastrointestinal oncology

## Abstract

Heterotopic pancreas (HP) is the presence of pancreatic tissue outside of its normal anatomical position without vascular continuity from the main pancreas. HP is most commonly found within the gastrointestinal tract, particularly the stomach through the jejunum. This report shares the case of a 57-year-old man who presented with persistent vomiting despite medical therapy. Given the nonspecific and broad differential diagnosis, a histopathological examination was warranted for a definitive diagnosis that showed a uniquely large and well-differentiated type I HP in the lesser curvature of the stomach. Resection was completed which was followed with resolution of symptoms.

## Introduction

Heterotopic pancreas (HP), also referred to as aberrant, accessory, or ectopic pancreas, is a condition in which pancreatic tissue is located outside of its normal anatomical position. The location of the HP varies, existing in any position within the abdominal cavity, but is commonly found within the stomach, duodenum, jejunum, and perigastric fat [1]. HP is widely considered a congenital anatomic abnormality. The incidence of HP has been reported as 0.18% in upper abdominal operations and at autopsies ranging from 0.6% to 14% indicating its asymptomatic nature [2]. Clinical presentation is often asymptomatic as most HP masses are found to be <1 centimeter [1]. The presence of symptoms varies based on the size and location of the ectopic tissue and is commonly associated with obstruction, inflammation, and/or malignant conversion. Some of the nonspecific symptoms include abdominal pain, distension, nausea and vomiting, malaise, anorexia, weight loss, and gastrointestinal bleeding [3]. Esophagogastroduodenoscopy (EGD) and endoscopic ultrasound (EUS) are used to identify locations for biopsy and fine-needle aspiration (FNA) for histologic evaluation. HP tissue is commonly found in the stomach and the size has been averaged to be less than 1 cm allowing it to oftentimes be asymptomatic. This case presents a patient who had persistent symptoms of vomiting and was found to have HP tissue in the stomach that was double the size of the average, which made it highly suspicious for a neoplastic process.

## Case presentation

A 57-year-old man with a past medical history of left neck squamous cell carcinoma (SCC) status post chemotherapy and radiation in remission has presented to the gastroenterology clinic complaining of a two-month history of throat pain and increasing frequency of early morning vomiting without nausea. He denied any recent medication change, abdominal pain, dysphagia to foods or liquids, unexplained weight loss, change in bowel movements, hematemesis, melena, or hematochezia. Earlier, the patient tried taking famotidine, omeprazole, and ondansetron with no improvement in symptoms.

**Figure 1:**
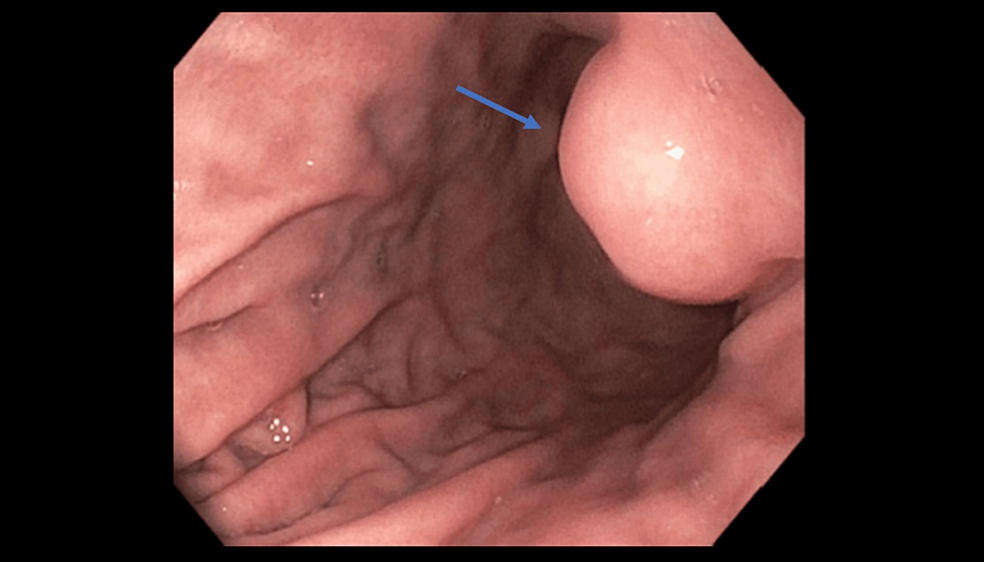
EGD showing a submucosal non-circumferential nodule (indicated by blue arrow) at the lesser curvature proximal to the incisura. EGD: esophagogastroduodenoscopy

Physical exam and routine laboratory findings were unremarkable. Computed tomography (CT) of the chest without contrast was negative for any acute findings. Because of the previous history of SCC of the head and neck, a positron emission tomography (PET) scan was also done to rule out any new metastatic findings which were also unremarkable at that time. EGD revealed the gastroesophageal (GE) junction at 35 cm from the incisors and was irregular. In addition, a medium-sized, submucosal non-circumferential mass without bleeding measuring 2x2 cm was found in the lesser curvature immediately proximal to the incisura with a completely normal appearing overlying mucosa (Figure 1). Results of EGD biopsies revealed gastric fundic-type mucosa with parietal cell hypertrophy and reflux esophagitis at the esophagogastric junction.

Due to his previous cancer history, a subsequent upper EUS was performed which revealed a 26x13 mm oval intramural (subepithelial) nodule in the incisura of the stomach. The nodule was cystic appearing with solid and liquid components originating from the submucosa. Due to high suspicion of malignancy considering a previous history of head and neck cancer, FNA was collected for evaluation. Pathology of the aspiration revealed smears of cellular clusters of monotonous cells, however, these clusters were not present on the cell block, so immunostains were unable to be performed, but neuroendocrine neoplasm could not be excluded. CT of the abdomen and pelvis was performed to evaluate the lesion position for surgical planning and nodal disease evaluation. The CT revealed a mixed cystic and solid lesion measuring 27x14 mm in biaxial dimension along gastric wall lesser curvature at the distal body antrum junction.

**Figure 2:**
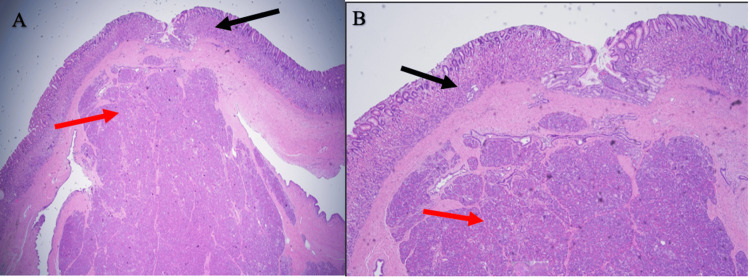
(A) H&E-stained sections show normal stomach mucosa on the top (black arrow) and pancreatic heterotopic tissue in the submucosa (red arrow) in 2X magnification and (B) in 4X magnification.

**Figure 3:**
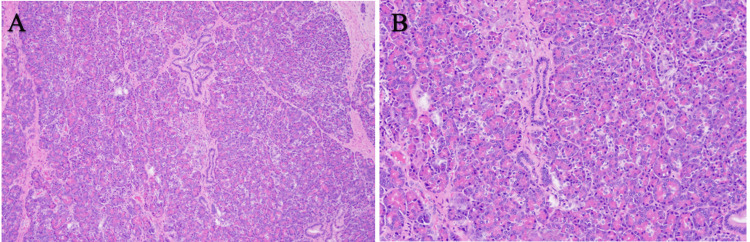
(A) Benign pancreatic acini and ducts show no significant pathologic abnormalities in 10X magnification and (B) in 20X magnification.

Due to the pathological findings concerning for neuroendocrine-type tumor, previous cancerous history, and persistent symptoms, the decision was made to remove the mass via robotic-assisted laparoscopic partial gastrectomy with intraoperative EGD and wedge resection for pathological interpretation. Microscopically, the mass was composed of unremarkable and uninflamed pancreatic tissue comprising acini, ducts, and islets of Langerhans cells, which was diagnosed as an HP. Hematoxylin and eosin (H&E) staining revealed normal superficial stomach mucosa; however, pancreatic heterotopic tissue was present in the submucosa (Figure 2). There was no evidence of atypia or neuroendocrine features (Figure 3). Upon a six-week follow-up, the patient reported a resolution of vomiting episodes.

## Discussion

HP is a rare anatomic abnormality that is most reported as an incidental finding during endoscopy, or in the resection or biopsy of specimens for other diseases [2]. Epidemiology of HP shows a peak incidence in the fourth and fifth decades of life with a slight female predominance. HP is identified most commonly in the stomach (52.7%), duodenum and proximal jejunum (25%), and perigastric fat (14%). HP lesions vary in size, however, most have been found to be less than 1 cm, with an average measurement of 0.84 cm [1]. The reported incidence of HP ranges from 0.18% [1] identified in operative or endoscopic procedures to as high as 0.6% to 14% [2]. The prevalent theory on the pathophysiology of HP is the misplacement theory, stipulating that pancreatic tissue is displaced from its origin during early development with foregut rotation [4].

Eighty percent of HP are asymptomatic, but clinically significant lesions may cause nonspecific symptoms such as abdominal pain, nausea, vomiting, diarrhea, and weight loss, likely related to larger size and associated obstruction and inflammation [5,2]. HP with functional components has been reported to cause acute and chronic pancreatitis with impaired drainage and obstruction of ducts. Severe inflammation and pseudocyst formation may occur even with modest elevations of enzymes. Gastric outlet obstruction has been observed in 9% of documented HP cases where these processes partially impeded gastric emptying. It has also been proposed that HP has the potential to cause a functional obstruction due to inducing pyloric spasm [6]. Both mechanisms have the potential to manifest as a patient complaint of vomiting.

It is difficult to visually distinguish HP endoscopically due to its small size and similar appearance to other gastrointestinal nodules. Other submucosal gastrointestinal nodules that could be considered on the differential diagnosis would be mesenchymal tumors (gastrointestinal stromal tumor (GIST), leiomyoma, granular cell tumor, lipoma, etc.), lymphoma tumors (mucosa-assisted lymphoid tissue (MALT) lymphoma, malignant lymphomas), epithelial tumors (neuroendocrine, carcinoma, etc.), congenital tumors (cyst, duplication, etc.) and others [7]. Thus, it is essential to have pathologic and histologic confirmation through biopsy to confirm the diagnosis of HP [8]. In addition, ectopic pancreatic tissue is typically located in the submucosal layer and therefore cannot be easily identified and sampled from the surface of the lesion. Histological examination is therefore essential for diagnosis. Tissue and cells for examination are obtained either by ultrasound-guided FNA or by biopsy during EGD or other surgery [4]. Histologic classification of ectopic pancreatic tissue was developed by Heinrich in 1909 and further modified by Gaspar-Fuentes in 1973. Type I, the most common, which was also found in our presenting case, consists of ectopic pancreatic tissue containing acini, ducts, and islets of Langerhans. Type II is composed of acini and pancreatic ducts without islets. Type III is composed of only ductal tissue, and type IV is composed of islet cells only [6].

Malignant transformation is rare, with an incidence rate ranging from 0.7% to 1.8%. Characteristics of HP that are commonly associated with malignancy include classification as a type I heterotopia by Heinrich classification, location in the stomach, ectopic tissue size greater than 4 cm, male gender, and middle age. The most common type of malignancy with such origin is adenocarcinoma (74%) followed by neuroendocrine tumors (7%), cystadenocarcinoma (6%), and pseudopapillary tumors (4%) [9]. Prognosis is reportedly difficult to determine due to the rarity of these tumors but is approximated to be better than that of a primary pancreatic cancer due to earlier presentation and identification [2].

Treatment options for HP range from observation of asymptomatic HP to resection of clinically significant HP. While malignant transformation is rare, excision of the mass via minimally invasive procedures is recommended for adequate histopathologic examination as well as to avoid future complications [2]. This case uniquely demonstrates a male patient who presented with daily morning vomiting lasting at least two months that was refractory to medical therapy. The patient was found to have a well-differentiated type I HP containing distinct acini, ducts, and islets within the wall of the stomach. The HP mass was unusually large (2x2 cm) requiring resection from the lesser curvature of the stomach proximal to the incisura to avoid malignant transformation and to provide symptomatic relief. Symptoms resolved with resection, conveying the clinical significance of this lesion. Symptomatic HP may indicate the presence of obstruction, inflammation, and/or malignant conversion, and as such this case illustrates a rare but important presentation of HP.

## Conclusions

This case presentation illustrates an unusually large HP as an uncommon cause of persistent vomiting. Clinical presentation is oftentimes asymptomatic; however, larger lesions can present with nonspecific symptoms such as abdominal pain, nausea, vomiting, diarrhea, and weight loss. Given the broad differential diagnosis for these nonspecific symptoms, EGD followed by EUS can be used to determine lesion, size, and location, and assist with image-guided tissue aspiration or biopsy to acquire the final diagnosis. Pathological analysis is required for diagnosing a gastric submucosal nodule with a broad differential diagnosis. H&E staining reveals ectopic pancreatic tissue components like acini, ducts, and/or islets of Langerhans. The preferred treatment is often resection as there is minimal scientific understanding of the prognostic risk of conversion to heterotopic pancreatic adenocarcinoma as done in this patient. Although HP is uncommon, it should be considered as a differential diagnosis in patients with non-specific symptoms like vomiting and unresponsive to medical therapy.
